# Design and Synthesis of Some New 1,3,4-Thiadiazines with Coumarin Moieties and Their Antioxidative and Antifungal Activity

**DOI:** 10.3390/molecules19011163

**Published:** 2014-01-17

**Authors:** Milan Čačić, Valentina Pavić, Maja Molnar, Bojan Šarkanj, Elizabeta Has-Schon

**Affiliations:** 1 Department of Applied Chemistry and Ecology, Faculty of Food Technology, J. J. Strossmayer University, Franje Kuhača 20, Osijek 31 000, Croatia; E-Mails: maja.molnar@ptfos.hr (M.M.); bojan.sarkanj@ptfos.hr (B.S.); 2 Department of Biology, J. J. Strossmayer University, Cara Hadrijana 8/A, Osijek 31 000, Croatia; E-Mails: valentina.pavic@biologija.unios.hr (V.P.); hasschon@biologija.unios.hr (E.H.-S.)

**Keywords:** coumarin, 1,3,4-thiadiazines, thiosemicarbazides, antifungal activity, antioxidative activity

## Abstract

A series of newly disubstituted (compounds **4a**,**b**) and trisubstituted 1,3,4-thiadiazines **5a**–**l** with various substituents was prepared utilizing different thiosemicarbazides and 3-α-bromoacetylcoumarins as starting compounds. The structures of the synthesized 1,3,4-thiadiazines are elucidated and confirmed utilizing the corresponding analytical and spectroscopic data. All of the new thiadiazine derivatives were tested for their antioxidant activity, employing different antioxidant assays (DPPH scavenging activity, iron chelating activity, power reducing activity). Compounds **5b**, **5f**, **5j** and **4b** were proven to be the best DPPH radical scavengers, while compounds **5h** and **5j** have shown the best iron chelating activity. Thiadiazine derivatives were also tested on their antifungal activity against four mycotoxicogenic fungi, *Aspergillus flavus, A. ochraceus, Fusarium graminearum* and *F. verticillioides*. The best antifungal against *A. flavus* was proven to be compound **5e**, while compounds **4a** and **5c** were the best antifungals on *A. ochraceus*, and compound **5g** showed the best antifungal activity on *F. verticillioides*.

## 1. Introduction

Coumarin (2-oxo-2*H*-chromene) and its derivatives represent one of the most important classes of compounds possessing numerous biological activities [1,2,3]. Some of these compounds have proven to be active as antibacterial [4,5,6], antifungal [7], anti-inflammatory [8], anticoagulant [9], anti-HIV [10] and antitumor agents [11]. Coumarin derivatives are widely used as additives in food and cosmetics [12], pharmaceuticals and optical brighteners [13] and laser dyes [14]. Coumarins have also proven to be an excellent antioxidants as well as antifungal agents [15].

In recent years, interest in thiadiazines has increased due to the high biological activity and broad-spectrum action of their derivatives [16]. Many thiadiazines have been discovered with possible applications in medical practice as sedatives, antianxiety agents, antiasthmatic agents, anticonvulsants, myorelaxants, coronary vasodilators, and spasmolytics. Synthesis of the 1,3,4-thiadiazine system employing a reaction of α-bromoacetophenone with thiosemicarbazide was first reported by Bose [17]. This procedure is characterized by the formation of a few heterocyclic isomers. Which isomer is formed depends on the H^+^-ion concentration in the system, the polarity of the solvent, the reaction temperature and on the substituents. Depending on whether condensation occurs at the N_1_, N_2_, or N_4_ of the thiosemicarbazide, three different sulfur-containing heterocyclic rings are expected after ring closure: the 6*H*-1,3,4-thiadiazines, the 2-substituted imino-2,3-dihydrothiazol-3-amines, and the 3-substituted 2-hydrazono-2,3-dihydrothiazoles.

In addition, 1,3,4-thiadiazines may exist in three different tautomeric forms. Spectroscopic investigations suggest that the 6H-form is preferred. The 4*H*-form represents a potentially anti-aromatic 8π-system which can be transformed by valence isomerization into a thiahomopyrazole and by subsequent extrusion of sulfur into a pyrazole [18]. In continuation of our research program on the synthesis of novel heterocyclic compounds exhibiting antioxidative and biological activity, it was thought to be interesting to synthesize compounds containing both coumarin and thiadiazine groups in their structure.

## 2. Results and Discussion

### 2.1. Chemistry

**Scheme 1:**
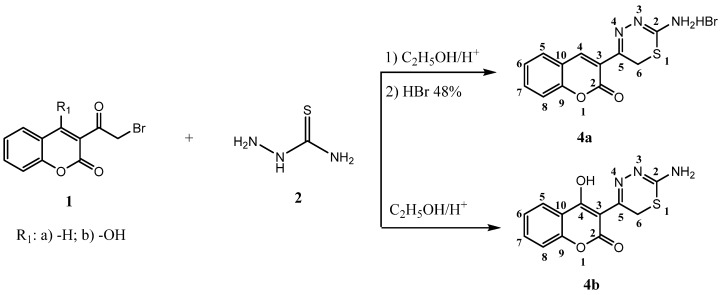
The reaction sequence for the synthesis of 5-(2-oxo-2*H*-chromen-3-yl)-6*H*-1,3,4-thiadizin-2-aminium bromide **(4a**) and 3-(2-amino-6*H*-1,3,4-thiadiazin-5-yl)-4-hydroxy-2*H*-chromen-2-one (**4b**).

In continuation of our ongoing research program to synthesize potentially biologically active 1,3,4-thiadiazine derivatives, in a present report we describe a series of 6*H*-1,3,4-thiadizines (compounds **4a**, **4b**) and 4*H*-1,3,4-thiadiazines (compounds **5a**–**l**). The reaction sequence for the synthesis of 5-(2-oxo-2*H*-chromen-3-yl)-6*H*-1,3,4-thiadizin-2-aminium bromide **(4a**) and 3-(2-amino-6*H*-1,3,4-thiadiazin-5-yl)-4-hydroxy-2*H*-chromen-2-one (**4b)**, is outlined in Scheme 1. 3-(α-Bromoacetyl)coumarins **1a,b** were prepared according to the earlier literature methods [19,20]. Reaction of **1a** with thiosemicarbazide **2** in ethanol at room temperature as the first step, followed by refluxing of the ethanolic solution of the obtained product in the presence of 48% HBr gave compound **4a**. Compound **4b** was prepared from **1b** and thiosemicarbazide in ethanol in a presence of small amount of HCl.

The structures of the products **4a** and **4b** were inferred from their analytical and spectral data. Thus, their IR spectra showed characteristic absorption bands at 3426–3183 (NH; OH), 1714–1696 (lactone C=O), 1617–1600 (-C=N), 756–760 (CH-arom.) and (C-S-C) at 675 cm^−1^. The ^1^H-NMR spectra of compounds **4a** and **4b** exhibited singlets at 3.91 (s, 2H, CH_2_), 4.80 (s, 2H, NH_2_/-N^+^H_3_) and 14.68 ppm (s, 1H, -OH).

1,4-Thiosemicarbazide derivatives **3a**–**g** were synthesized as previously described [21] by reacting equimolar amounts of ethyl-, methyl- or phenylisothiocyanate with 4-hydroxybenzohydrazide, 2-(7-hydroxy-2-oxo-2H-chromen-4-yl)acetohydrazide and 2-(4-methyl-2-oxo-2*H*-chromen-7-yloxy)-acetohydrazide.

**Scheme 2:**
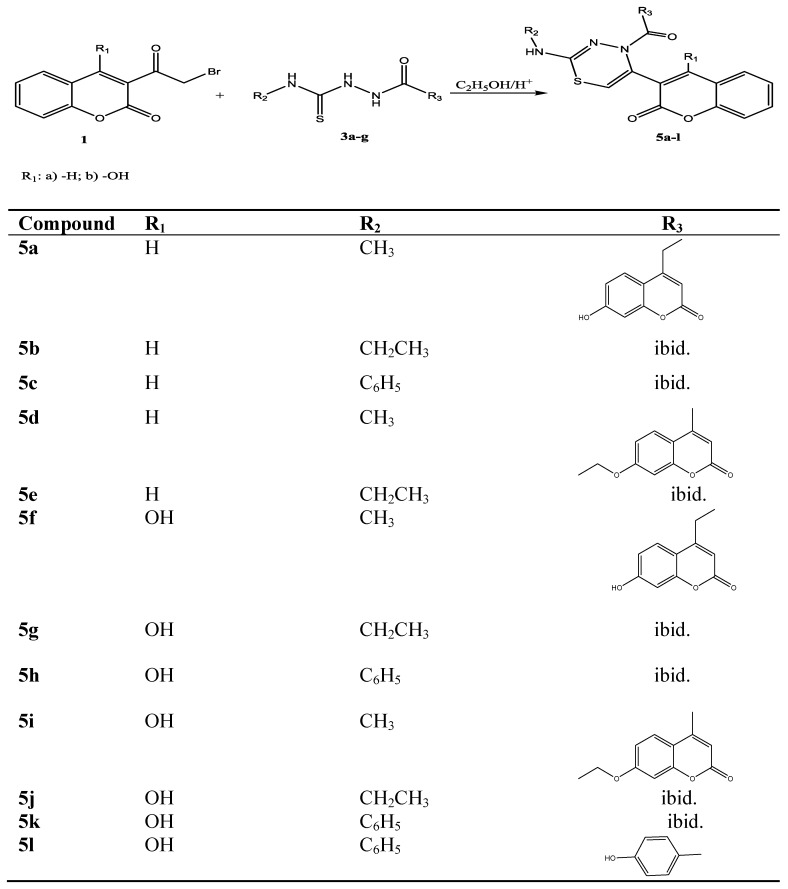
Synthetic path for compounds **5a**–**l**.

The compounds **5a**–**l** were prepared by heating 3-α-bromoacetylcoumarins **1a**,**b** with the corresponding 1,4-disubstituted thiosemicarbazides **3a**–**g** in ethanol, with few drops of hydrochloric acid for about 40 min (Scheme 2).

Newly synthesised compounds **5a**–**l** were characterised on the basis of elemental analysis, IR, ^1^H NMR and mass spectral data. The IR spectra of compounds **5a**–**l** showed absorption bands in the range from 3490–2920 cm^−1^ due to –CH_2_- stretching, C-H arom. and NH. The strong band at 1740 and band at 1690 cm^−1^ is attributed to the C=O stretching vibration. The absorption band seen at a 1600–1560 cm^−1^ could be attributed to the C=N stretching. The weak absorption band showed in the range 681–639 cm^−1^ is attributed to the (C-S-C) stretching vibration.

The ^1^H-NMR spectra of **5a**–**l** showed singlet in the range 5.56–5.67 ppm, which is characteristic for S-CH=C- from 1,3,4-thiadiazine ring. The combined spectral data gave strong support to the proposed structures of all the synthesized compounds. 

### 2.2. DPPH-Scavenging Activity

The DPPH free radical exhibits a strong absorption maximum at 517 nm, which is purple in color. Upon the addition of an antioxidant a reduction of DPPH radical to DPPH-H occurs and the color turns from purple to yellow. The method used in this work determines the antioxidant capacity by measuring the remaining amount of DPPH after 30 min of incubation. This DPPH-scavenging activity was compared to ascorbic acid as a standard compound.

As it can be seen from Table 1, the thiadiazine derivatives are excellent antioxidants. The best ones are compounds **5b**, **5f**, **5j** and **4b**, possessing DPPH scavenging activity in a range of ascorbic acid. All of these compounds incorporate a -OH group in their structure—**5f**, **5j** and **4b** in position 4 of coumarin core and **5b** on the coumarin core linked to the 1,3,4-thaidiazine moiety of the compound. Compounds **5g**, **5e**, **5h** and **5l** also exhibit very high DPPH scavenging activity, in a range 80%–90%, but lower than that of the compounds mentioned above. From their structure it is evident that they all, except for compound **5e**, bear two hydroxyl groups in their structure. Compounds **5i** and **5k** exhibit DPPH scavenging activity >70%, and the other compounds did not show significant activity. Since these compounds are complex structures, most of them even containing two coumarin cores, their activity is probably highly dependent on their stereochemistry.

**Table 1:** DPPH scavenging activity and iron chelating activity of thiadiazine derivatives ^a^.

Compound	% DPPH scavenging activity	% Chelating activity
ascorbic acid ^b^	85.2 ± 6.1	-
EDTA ^c^	-	90.9 ± 7.22
4a	17.2 ± 0.59	0.0
4b	90.0 ± 1.24	15.1 ± 4.27
5a	36.0 ± 2.07	-
5b	94.4 ± 1.73	0
5c	17.7 ± 1.26	0,0
5d	33.3 ± 0.27	-
5e	80.7 ± 1.61	0.0
5f	94.1 ± 1.35	0.0
5g	82.3 ± 0.70	0.0
5h	80.6 ± 0.98	62.5 ± 2.25
5i	79.7 ± 1.29	5.3 ± 0.56
5j	90.0 ± 1.08	53.5
5k	77.2 ± 0.93	5.18 ± 0.81
5l	80.2 ± 0.44	10.2 ± 0.42

^a^ data are means ± standard deviation of three replicates; ^b^ ascorbic acid was used as standard in DPPH scavenging activity determination; ^c^ EDTA was used as standard in iron chelating activity determination.

### 2.3. Iron Chelating Activity

This assay is based upon the complex formation between ferrozine and Fe^2+^, which is red in color. In the presence of chelating agents, the complex formation is disrupted and the red color of the complex is decreased, which can be monitored by spectrophotometric measurement of the color reduction at 562 nm.

Iron is known to be one of the most important lipid oxidation prooxidants due to its high reactivity and may participate in hydroxyl radical generating Fenton type reactions. Thus effective ferrous ion chelators may be important in protection against oxidative damage by removing ferrous ion (Fe^2+^) [22]. The most potent iron chelators amongst thiadiazine derivatives investigated in our research are shown to be compounds **5h** and **5j**, while other compounds did not show significant activity.

### 2.4. Reducing Power

**Figure 1:**
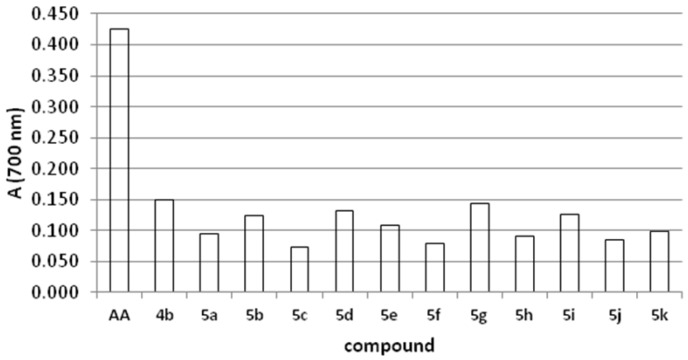
Reducing power of tested thiadiazine derivatives.

This assay is based on the reduction of Fe^3+^ from potassium ferricyanide to Fe^2+^ (potassium ferrocyanide), which then reacts with ferric chloride to form a ferric/ferrous complex with an absorption maximum at 700 nm [23,24]. A higher absorbance indicates better reducing power of the compound. Compounds possessing the highest reducing power in this assay were **4b**, **5g**, **5d**, but their activity was not comparable to that of ascorbic acid, which was used as standard compound (Figure 1).

### 2.5. Antifungal Activity

The antifungal activity of thiadiazine derivatives strongly depends on the fungal species. The best antifungal agent on *A. flavus* (Table 2) was proven to be compound **5e**, while observing the overall antifungal activity results, compared to other molds *A. flavus* was the most resistant to the tested compounds. 

**Table 2:** Minimal inhibitory concentration for 50% cell death for the tested thiadiazine derivatives.

MIC_50_				
Compound	*A. flavus*	*A. ochraceus*	*F. graminearum*	*F. verticillioides*
**4a**	/	0.01	0.01	0.1
**5a**	1	0.1	0.1	0.1
**5b**	/	0.1	0.01	0.1
**5c**	0.1	0.01	0.01	0.1
**5d**	0.1	0.1	0.01	1
**5e**	0.01	0.1	0.01	0.1
**5f**	0.1	0.1	0.01	0.1
**5g**	1	0.1	0.01	0.01
**5i**	1	0.1	0.01	1
**5j**	1	0.1	0.01	0.1

Compounds **4a** and **5c** were the best antifungals on *A. ochraceus*, and compound **5g** showed the best antifungal activity on *F. verticillioides*. *F. graminearum* was the most susceptible mold to the tested compounds and most of the compounds showed high antifungal activity towards this mold. This kind of behavior was also observed in our previous research [15], which could be important since this mold was found to be a potential threat in production of malt barley and wheat in our region [25]. 

## 3. Experimental

### 3.1. General

Melting points were determined on a capillary melting point apparatus (Electrotermal, Rochford, Great Britain) and are uncorrected. Thin-layer chromatography was performed with fluorescent silica gel plates HF_254_ (Merck, Darmstadt, Germany), which were checked under UV (254 and 365 nm) light, using benzene:acetone:acetic acid (8:1:1) as a solvent. The elemental analysis for C, H and N was done on a Analyzer 2440 (Perkin–Elmer, Boston, MA, USA). Infrared spectra (ν_max_/cm^−1^) were recorded on a FT-IR 3303 spectrometer (Beckman Instruments, Inc., Irvine, CA, USA), using KBr disks. ^1^H-NMR spectra were recorded on a Bruker Avance 600 MHz NMR Spectrometer (Bruker Biospin GmbH, Rheinstetten, Germany) at 293 K in DMSO-d6. The MS spectra were recorded on LC/MS/MS API 2000 (Applied Biosystems/MDS SCIEX, Foster City, CA, USA). The absorbance was measured on a UV visible spectrophotometer Heliosγ, (Thermo Spectronic, Cambridge, UK). Microplates were read on a Sunrise absorbance reader (Tecan Group Ltd., Männedorf, Switzerland). Incubation was carried in an Aqualytic AL 500-8 incubator (Aqualytic, Dortmund, Germany). 

### 3.2. Synthesis

#### 3.2.1. General Procedure for the Preparation of 1,4-Disubstituted Thiosemicarbazides **3** [21]

To a solution of corresponding carbohydrazide (1 mmol) in ethanol (5–10 mL), alkyl/aryl isothiocyanate (1 mmol) and sodium hydroxide (40 mg, 1 mmol, as a 2N solution) were added. The mixture was refluxed for 2–4 h. The precipitate was filtered and crystallized from ethanol/water, to give compounds **3a**–**g** in 65%–85% yield.

#### 3.2.2. Synthesis of 5-(2-Oxo-2*H*-chromen-3-yl)-6*H*-1,3,4-thiadizin-2-aminium Bromide (**4a**)

A mixture of 3-α-bromoacetylcoumarin (1a, 0.01 mol) and thiosemicarbazide (0.01 mol) was suspended in ethanol (35 mL) at 0 °C. The mixture was allowed to warm up to room temperature overnight under stirring. The resulting slurry was cooled to −18 °C and the precipitate was collected by filtration, washed with cold ethanol, and dried under vacuum. The yellow solid was again suspended in ethanol (25 mL) which contained 48% aqueous hydrobromic acid (1 mL). The reaction mixture was heated to reflux for 45 min. and then allowed to cool down overnight to room temperature. The precipitate was filtered, recrystallized from ethanol, and dried over phosphorus pentoxide under high-vacuum at 40 °C. Yellow crystals (70% yield); mp 240 °C, R_f_ = 0.52; FT-IR ν_max_ 3426, 3183, 1714, 1696, 1617, 1557, 1451, 1367, 1250, 1179, 965, 841, 756 and 675 cm^−1^; ^1^H-NMR (DMSO-*d*_6_) *δ* (ppm): 3.91 (s, 2H, CH_2_), 4.80 (s,2H, NH_2_/-N^+^H_3_), 7.02–7.27 (m, 4H, coum.), 7.45 (s, 1H, H-4 coum); MS *m/z* 339.0 [M-H]^+^, (M = 340.2); Anal. Calcd. for C_12_H_10_BrN_3_O_2_S: C, 42.37; H, 2.96; N, 12.35%; Found: C, 42.39; H, 2.94; N, 12.37%.

#### 3.2.3. Synthesis of 3-(2-Amino-6*H*-1,3,4-thiadiazin-5-yl)-4-hydroxy-2H-chromen-2-one (**4b**)

Compound **4b** was prepared by heating of 3-α-bromoacetylcoumarin (**1b,** 0.01 mol) with thiosemicarbazide (0.01 mol) in ethanol (40 mL) with a few drops of hydrochloric acid for 20 min. The reaction mixture was cooled and adjusted to pH 8-9 by adding a dilute solution of ammonia. A brown colored precipitate was formed and filtered off, then recrystallized from 30% ethanol, and dried under high-vacuum at 40 °C over phosphorus pentoxide. Pale brown crystals (75% yield); mp 210 °C; R_f_ = 0.66; FT-IR ν_max_ 3416, 3143, 1687, 1656, 1571, 1557, 1410, 1257, 1110, 965, 891, 758 and 678 cm^−1^. ^1^H-NMR (DMSO-*d*_6_) *δ* (ppm): 3.91 (s, 2H, CH_2_), 4.2 (s, 2H, NH_2_), 7.02–7.27 (m, 4H, coum.), 7.45 (s, 1H, H-4 coum.), 14.68 (s, 1H, -OH); MS *m/z* 274.01[M–H]^+^, (M = 275.04); Anal. Calcd. for C_12_H_9_N_3_O_3_S: C, 52.36; H, 3.30; N, 15.26%; Found: C, 52.39; H, 3.29; N, 15.24%.

#### 3.2.4. General Procedure for Preparation 2,4,5-Trisubstituted 4*H*-1,3,4- thiadiazines **5a**–**l**

Compounds **5a**–**l** were prepared by heating 3-α-bromoacetylcoumarin **1a**,**b** (0.01 mol) with the corresponding 1,4-disubstituted thiosemicarbazide **3a**–**g** (0.01 mol) in ethanol (40 mL) with a few drops of hydrochloric acid for 40 min. The reaction mixture was cooled and made alkaline with a diluted solution of ammonia to adjust the pH to 8–9. The precipitate formed was filtered off, recrystallized from 30% water-ethanol, and dried under high-vacuum at 40 °C over phosphorus pentoxide.

*3-(4-(2-(7-Hydroxy-2-oxo-2H-chromen-4yl)acetyl)-2-(methylamino)-4H-1,3,4-thiadiazin-5-yl)-2H-chromen-2-one* (**5a**). Brown crystals (48% yield); mp 262 °C; R_f_ = 0.56; FT-IR ν_max_ 3227, 2979, 1710, 1682, 1607, 1560, 1478, 1395, 1330, 1209, 1139, 959, 843,759 and 639 cm^−1^; ^1^H NMR (DMSO-*d*_6_) *δ* (ppm): 2.72 (d, 3H, CH_3_), 3.85 (s, 2H, CH_2_), 4.80 (q, 1H, NH), 5.56 (s, 1H, thiadiaz.), 6.04–7.37 (m, 9H, coum. 10.86 (s, 1H, OH), MS *m/z* 475.1[M–H]^+^, (M=475.5); Anal. Calcd. for C_24_H_17_N_3_O_6_S: C, 60.63; H, 3.60; N, 8.84; Found: C, 60.59; H, 3.60; N, 8.81%.

*3-(2-(Ethylamino)-4-(2-(7-hydroxy-2-oxo-2H-chromen-4yl)acetyl)-4H-1,3,4-thiadiazin-5-yl)-2H-chromen-2-one* (**5b**). Brown crystals (52% yield); mp 194 °C; R_f_ = 0.54; FT-IR ν_max_ 3492, 3134, 2978, 1708, 1673, 1607, 1566, 1552, 1478, 1392, 1210, 1062, 854, 759 and 639 cm^−1^; ^1^H-NMR (DMSO-*d*_6_) *δ* (ppm): 2.42 (t, 3H, CH_3_), 2.69 (q, 2H, CH_2_), 2.85 (s, 2H, CH_2_), 4.90 (t, 1H, NH), 5.0 (s, 1H, OH), 5.60 (s, 1H, thiadiaz.), 6.04–7.37 (m, 9H, coum.), 11.20 (s, 1H, OH); MS *m/z* 489.1[M-H]^+^, (M = 489.5); Anal. Calcd. for C_25_H_19_N_3_O_6_S: C, 61.34; H, 3.91; N, 8.58; Found: C, 61.32; H, 3.89; N, 8.53%.

*3-(4-(2-(7-Hydroxy-2-oxo-2H-chromen-4yl)acetyl)-2-(phenylamino)-4H-1,3,4-thiadiazin-5-yl)-2H-chromen-2-one* (**5c**). Pale grey crystals (62% yield); mp 252 °C; R_f_ = 0.51; FT-IR ν_max_ 3350, 3231, 3052, 1733, 1691, 1608, 1570, 1457, 1395, 1310, 1136, 1095, 996, 841, 759 and 693 cm^−1^; ^1^H-NMR (DMSO-*d*_6_) *δ* (ppm): 2.85 (s, 2H, CH_2_), 4.6 (s, 1H, NH), 5.67 (s, 1H, thiadiaz.), 6.04–7.37 (m, 14H, coum. and arom.), 10.52 (s, 1H, OH); MS *m/z* 537.1[M-H]^+^, (M = 537.5); Anal. Calcd. for C_29_H_19_N_3_O_6_S: C, 64.80; H, 3.56; N, 7.82; Found: C, 64.79; H, 3.52; N, 7.80%.

*7-(2-(2-(Methylamino)-5-(2-oxo-2H-chromen-2-oxoethoxy)-3-yl)-4H-1,3,4-thiadiazin-4-yl)-4-methyl-2H-chromen-2-one* (**5d**). Pale grey crystals (72% yield); mp 205 °C; R_f_ = 0.51; FT-IR ν_max_ 3435, 3075, 2970, 2830, 1715, 1677, 1610, 1558, 1449, 1391, 1284, 1213, 1183, 1080, 982, 852, 765 and 684 cm^−1^; ^1^H-NMR (DMSO-*d*_6_) *δ* (ppm): 1.71 (s, 3H, CH_3_), 2.74 (d, 3H, NCH_3_), 4.83 (s, 2H, OCH_2_), 5.1 (q, 1H, NH), 5.64 (s, 1H, thiadiaz.), 5.90–7.28 (m, 8H, coum.); MS *m/z* 488.1[M-H]^+^, (M = 489.5); Anal. Calcd. for C_25_H_19_N_3_O_6_S: C, 61.34; H, 3.91; N, 8.58; Found: C, 61.32; H, 3.90; N, 8.53%.

*7-(2-(2-(Ethylamino)-5-(2-oxo-2H-chromen-3-yl)-4H-1,3,4-thiadiazin-4-yl)-2-oxoethoxy)-4-methyl-2H-chromen-2-one* (**5e**). Pale grey crystals (73% yield); mp 256 °C; R_f_ = 0.52; FT-IR ν_max_ 3434, 3174, 3029, 2734, 1765, 1715, 1607, 1556, 1449, 1391, 1277, 1153, 1072, 852, 759 and 681 cm^−1^; ^1^H-NMR (DMSO-*d*_6_) *δ* (ppm): 1.20 (t, 3H, CH_3_), 1.74 (s, 3H, CH_3_), 2.69 (q, 2H, CH_2_), 4.83 (s, 2H, OCH_2_), 5.21(q, 1H, NH), 5.60 (s, 1H, thiadiaz.), 5.90–7.28 (m, 9H, coum.); MS *m/z* 502.1[M-H]^+^, (M = 503.5); Anal. Calcd. for C_26_H_21_N_3_O_6_S: C, 62.02; H, 4.20; N, 8.35; Found: C, 61.92; H, 4.22; N, 8.30%.

*4-Hydroxy-3-(4-(2-(7-hydroxy-2-oxo-2H-chromen-4-yl)acetyl)-2-(methylamino)-4H-1,3,4-thiadiazin-5-yl)-2H-chromen-2-one* (**5f**). Pale yellow crystals (68% yield); mp 222 °C; R_f_ = 0.44; FT-IR ν_max_ 3449, 3337, 1707, 1692, 1613, 1560, 1448, 1395, 1269, 1139, 1032, 853, 759 and 693 cm^−1^; ^1^H-NMR (DMSO-*d*_6_) *δ* (ppm): 2.70 (d, 3H, CH_3_), 2.85 (s, 2H, CH_2_), 4.89 (q, 1H, NH), 5.65 (s, 1H, thiadi.), 6.04–7.28 (m, 8H, coum.), 10.80 (s, 1H, OH-7 coum), 15.0 (s, 1H, OH-4 coum); MS *m/z* 490.1[M-H]^+^, (M = 491.5); Anal. Calcd. for C_24_H_17_N_3_O_7_S: C, 58.65; H, 3.49; N, 8.55; Found: C, 58.62; H, 3.52; N, 8.53%.

*4-Hydroxy-3-(4-(2-(7-hydroxy-2-oxo-2H-chromen-4-yl)acetyl)-2-(ethylamino)-4H-1,3,4-thiadiazin-5-yl)-2H-chromen-2-one* (**5g**). Pale yellow crystals (72% yield); mp 261 °C; R_f_ = 0.53; FT-IR ν_max_ 3447, 3413, 2986, 1708, 1688, 1615, 1562, 1550, 1392, 1269, 1139, 1032, 829, 758 and 690 cm^−1^; ^1^H-NMR (DMSO-*d*_6_) *δ* (ppm): 1.30 (t, 3H, CH_3_), 2.69 (q, 2H, CH_2_), 2.85 (s, 2H, CH_2_), 5.21 (t, 1H, NH), 5.63 (s, 1H, thiadiaz.), 6-04-7.27 (m, 8H, coum.), 10.86 (s, 1H, OH-7 coum), 15.1 (s,1H, OH-4 coum); MS *m/z* 504.1[M-H]^+^, (M = 505.5); Anal. Calcd. for C_25_H_19_N_3_O_7_S: C, 59.40; H, 3.79; N, 8.31; Found: C, 59.42; H, 3.82; N, 8.34%.

*4-Hydroxy-3-(4-(2-(7-hydroxy-2-oxo-2H-chromen-4-yl)acetyl)-2-(phenylamino)-4H-1,3,4-thiadiazin-5-yl)-2H-chromen-2-one* (**5h**). Pale yellow crystals (85% yield); mp 289 °C; R_f_ = 0.65; FT-IR ν_max_ 3418, 3184, 1703, 1608, 1548, 1511, 1393, 1322, 1269, 1139, 1034, 852, 758 and 678 cm^−1^; ^1^H-NMR (DMSO-*d*_6_) *δ* (ppm): 2.85 (s, 2H, CH_2_), 5.62 (s, 1H, thiadiaz.), 6.04–7.27 (m, 13H, coum. and arom.), 8.11 (s, 1H, NH), 15.0 (s, 1H, OH); MS *m/z* 552.1[M-H]^+^, (M = 553.5); Anal. Calcd. for C_29_H_19_N_3_O_7_S: C, 62.92; H, 3.46; N, 7.59; Found: C, 62.89; H, 3.42; N, 7.53%.

*4-Hydroxy-3-(4-(2-(4-methyl-2-oxo-2H-chromen-7-yloxy)acetyl)-2-(methylamino)-4H-1,3,4-thia-diazin-5-yl)-2H-chromen-2-one* (**5i**). Pale yellow crystals (68% yield); mp 222–223 °C; R_f_ = 0.67; FT-IR ν_max_ 3496, 2674, 1715, 1696, 1614, 1554, 1540, 1394, 1266, 1211, 1148, 867, 759 and 682 cm^−1^; ^1^H NMR (DMSO-*d*_6_) *δ* (ppm): 1.71 (s, 3H, CH_3_), 2.73 (d, 2H, CH_3_-N), 4.83 (s, 2H, OCH_2_), 5.20 (q, 1H, NH), 5.66 (s, 1H, thiadi.), 5.90–7.28 (m, 8H, coum.), 10.86 (s, 1H, OH-7 coum), 14.6 (s, 1H, OH-4 coum), MS *m/z* 505.1[M-H]^+^, (M = 505.5); Anal. Calcd. for C_25_H_19_N_3_O_7_S: C, 59.40; H, 3.79; N, 8.31; Found: C, 59.32; H, 3.81; N, 8.30%.

*4-Hydroxy-3-(4-(2-(4-methyl-2-oxo-2H-chromen-7-yloxy)acetyl)-2-(ethylamino)-4H-1,3,4-thiadiazin-5-yl)-2H-chromen-2-one* (**5j**). Pale yellow crystals (78 % yield); mp 226 °C; R_f_ = 0.69; FT-IR ν_max_ 3433, 3169, 2707, 1723, 1676, 1611, 1553, 1492, 1391, 1267, 1210, 1143, 1070, 988, 841, 758 and 681 cm^−1^; ^1^H-NMR (DMSO-*d*_6_) *δ* (ppm): 1.24 (s, 3H, CH_3_), 1.74 (t, 3H, CH_3_), 2.0 (s, 1H, NH), 2.69 (q, 2H, CH_2_), 4.83 (s, 2H, OCH_2_), 5.31 (s, 1H, NH), 5.67 (s, 1H, thiadi.), 5.90–7.28 (m, 8H, coum.), 15.0 (s, 1H, OH-4 coum); MS *m/z* 518.1[M-H]^+^, (M = 519.5); Anal. Calcd. for C_26_H_21_N_3_O_7_S: C, 60.11; H, 4.07; N, 8.09; Found: C, 60.12; H, 4.02; N, 8.10%.

*4-Hydroxy-3-(4-(2-(4-methyl-2-oxo-2H-chromen-7-yloxy)acetyl)-2-(phenylamino)-4H-1,3,4-thia-diazin-5-yl)-2H-chromen-2-one* (**5k**). Pale yellow crystals (52% yield); mp 237–239 ° C; R_f_ = 0.45; FT-IR ν_max_ 3466, 3424, 3064, 2920, 1707, 1651, 1612, 1543, 1496, 1448, 1387, 1266, 1151, 1075, 842, 755 and 697 cm^−1^; ^1^H-NMR (DMSO-*d*_6_) *δ* (ppm): 1.71 (s, 3H, CH_3_), 4.83 (s, 2H, OCH_2_), 5.0 (q, 1H, NH), 5.60 (s, 1H, thiadiaz.), 5.90–7.27 (m, 13H, coum. and arom.), 15.1 (s, 1H, OH); MS *m/z* 566.1[M-H]^+^, (M = 567.6); Anal. Calcd. for C_30_H_21_N_3_O_7_S: C, 63.49; H, 3.73; N, 7.40; Found: C, 63.52; H, 3.62; N, 7.32%.

*4-Hydroxy-3-(4-(4-hydroxybenzoyl)-2-(phenylamino)-4H-1,3,4-thiadiazin-5-yl)-2H-chromen-2-one* (**5l**). Ppale yellow crystals (72% yield); mp 285 °C; R_f_ = 0.69; FT-IR ν_max_ 3445, 3224, 1753, 1693, 1612, 1560, 1532, 1438, 1258, 1103, 963, 845, 756 and 668 cm^−1^; ^1^H-NMR (DMSO-*d*_6_) *δ* (ppm): 5.20 (s, 1H, NH), 5.66 (s, 1H, thiadi.), 6.46–7.78 (m, 13H, coum. and arom.), 8.90 (s, 1H, OH), 15.0 (s, 1H, OH-4 coum.); MS *m/z* 469.81[M-H]^+^, (M = 471.5); Anal. Calcd. for C_25_H_17_N_3_O_5_S: C, 63.69; H, 3.63; N, 8.91; Found: C, 63.62; H, 3.42; N, 8.70%.

### 3.3. DPPH-Scavenging Activity

Determination of antioxidant activity was performed according to the procedure described in the literature [26,27] with some modifications described in our previous work [15]. Dimethyl sulfoxide (DMSO) was used as a solvent [28], due to the low solubility of synthesized compounds in ethanol and methanol. A DMSO solution (750 µL) of the corresponding thiadiazine derivative (0.2 mM) was added to a DMSO solution of DPPH radical (0.2 mM), so that the final concentration of DPPH radical and the synthesized compound in a solution was 0.1 mM. The mixture was shaken and allowed to stand at room temperature. After 30 min the absorbance at 517 nm was determined and the scavenging activity was calculated according to the formula below:

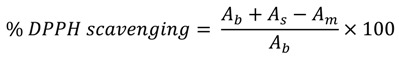
(1)
where A_b_–absorbance of 0.1 mM DMSO solution of DPPH radical at 517 nm; A_s_ – absorbance of 0.1 mM DMSO solution of test compound at 517 nm; A_m_ – absorbance of DMSO mixture of test com-pound and DPPH radical at 517 nm. Ascorbic acid (AA) was used as a reference compound.

### 3.4. Iron Chelating Activity

The chelating activity of thiadiazine derivatives for ferrous ions Fe^2+^ was measured according to the literature method [29] with some modifications. FeCl_2_ (2 mM, 25 µL) were added to 2 mM methanol/DMSO solution (1 mL, 4:1) of the compound investigated. After 30 s, ferrozine (5 mM, 50 µL) was added. Samples were incubated at room temperature for 10 min and the absorbance of the complex formed between Fe^2+^ and ferrozine was measured at 562 nm. Metal chelating efficiency of samples was compared to the chelating activity of EDTA disodium salt. The chelating activity of the extract for Fe^2+^ was calculated as:

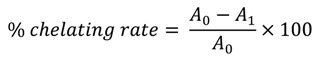
(2)
A_0_ – absorbance of the control (blank, without samples) at 562 nm; A_1_ – absorbance in the presence of the methanol/DMSO sample solution at 562 nm.

### 3.5. Reducing Power

The reducing power of thiadiazine derivatives was determined according to the method [30]. An aliquot of the 0.2 mM thiadiazine derivatives in DMSO (250 µL), were mixed with 0.2 M phosphate buffer (250 µL, pH 6.6) and potassium ferricyanide (K_3_Fe(CN)_6_, 250 µL, 1% w/v). The mixture was incubated at 50 °C for 20 min, followed by the addition of trichloroacetic acid (TCA, 250 µL, 10% w/v), centrifuged for 10 min at 2000 *×g*. A portion of the upper layer of the solution (300 µL) was mixed with distilled water (300 µL) and FeCl_3_ (0.5 mL, 0.1% w/v). The absorbance was measured at 700 nm. The higher absorbance indicates stronger reducing power of compound.

### 3.6. Antifungal Activity

#### 3.6.1. General

Broth microdilution assays were performed in accordance with the guidelines detailed in CLSI document M38-A [31].

#### 3.6.2. Tested Fungi

The fungi used in this experiment (*Aspergillus flavus* (NRRL 3251); *Aspergillus ochraceus* (CBS 589.68), *Fusarium graminearum* (CBS 110.250) and *Fusarium verticillioides* (CBS 119.825)) are the major producers of mycotoxins and food contaminants [32]. 

#### 3.6.3. Preparation of Inoculum

The inocula were obtained from cultures that were grown on potato dextrose agar (PDA) slants. *Aspergillus* strains were grown at 35 °C for 7 days, and *Fusarium* strains were grown at 35 °C for 2 days, followed by 7 days at 25 °C. Fungal spores were harvested by pouring 5 ml of sterile saline onto the culture slants and scraping the surface with an inoculation loop. The number of spores in the stock suspension was adjusted to 10^6^ spores/mL, using a Bürker–Türk counting chamber (Haemocytometer). Turbidity of each stock suspension was checked with a 0.5 McFarland standard read at 530 nm. Working 2× spore suspension was prepared by diluting 200 µL of stock suspension into 10 mL of RPMI 1640 medium [31]. 

#### 3.6.4. Medium

Antifungal susceptibility testing was performed, using RPMI 1640 medium buffered with 0.164 M MOPS (34.53 g/L) and adjusted to pH 7.0 with (1 M) NaOH, as recommended by the National Committee for Clinical Laboratory Standards. Medium was sterilized by filtration using a 0.45 µm filter.

#### 3.6.5. Drug Dilutions

The drug dilutions were prepared following the additive two fold drug dilution scheme described in NCCLS M28-A method for water-insoluble compounds [31]. Briefly, stock drug solutions were first diluted by 20× of the final concentrations in 100% dimethyl sulfoxide (DMSO), followed by further dilutions to the final drug concentrations, 10, 1, 0.1 and 0.01 µg/mL. All drug dilutions were sterilized by filtration using a 0.45 µm filter. Sterile polypropylene microtitre plates (96 U-shaped wells; Brand, Wertheim, Germany) were used in the microdilution test. Rows 2–11 contained the series of drug dilutions in 100 µL volumes and 100 µL of the 2× spore suspension. Row 1 contained 200 µL of uninoculated, drug-free medium and served as the sterility control, while row 12 contained 100 µL of drug-free medium and 100 µL of inoculum and served as growth control (the final volume in each well was 200 µL).

#### 3.6.6. Incubation and MIC Determination

Following inoculation, all plates were incubated at 35 °C in an atmospheric incubator. After 48 h of incubation, plates were read on a microplate reader at 450 nm. Minimal inhibitory concentration for 50% cell death (MIC 50) was defined as the lowest concentration reducing the optical density by 50% at 450 nm compared with growth control [33,34].

## 4. Conclusions

In this paper, and as a part of our ongoing research on biologically active heterocyclic compounds, some thiadiazine derivatives were successfully synthesized and characterized by various spectral methods. Compounds were subjected to antioxidant (DPPH scavenging, iron chelating and reducing power) and antifungal investigation on four mycotoxicogenic fungi, *A. flavus*, *A. ochraceus*, *F. graminearum* and *F. verticillioides* namely. The new thiadiazine derivatives were proven to possess an excellent antioxidant activity, comparable to ascorbic acid, while not showing reducing power activity at the same time. Also, most of the compounds were proven to possess potent antifungal activity towards all the tested fungi. Compounds showing the best activity have been selected for further investigation employing some *in vivo* models.
